# Prevalence of Swimming Puppy Syndrome in 2,443 Puppies during the Years 2006–2012 in Thailand

**DOI:** 10.1155/2013/617803

**Published:** 2013-05-28

**Authors:** Korakot Nganvongpanit, Terdsak Yano

**Affiliations:** ^1^Animal Bone and Joint Research Laboratory, Department of Veterinary Biosciences and Public Health, Faculty of Veterinary Medicine, Chiang Mai University, Chiang Mai 50100, Thailand; ^2^Materials Science Research Center, Faculty of Science, Chiang Mai University, Chiang Mai 50200, Thailand; ^3^Department of Food Animals, Faculty of Veterinary Medicine, Chiang Mai University, Chiang Mai 50100, Thailand

## Abstract

The purpose of this study was to report on the prevalence of swimming puppy syndrome (SPS) and investigate predisposing factors. Data were recorded from 2,443 puppies (1,183 males and 1,260 females) in Thailand, October 2006–September 2012, including breed, sex, number of puppies per litter, type of nest floor, number of affected limbs, and occurrence of pectus excavatum. Fifty-two puppies (2.13%) were diagnosed with SPS. The breed most frequently affected was English Bulldog (8.33%). There was no significant difference (*P* > 0.05) between presence and absence of disease based on sex, breed, and nest floor type. The number of puppies per litter was associated with SPS; puppies from smaller litters (1.92 ± 1.12) had a higher prevalence of the disease (*P* < 0.01) than puppies from larger litters (3.64 ± 2.24). Moreover, 15.38% of puppies with affected limbs showed signs of pectus excavatum (8/52); this clinical sign was more prevalent (*P* < 0.01) in puppies with all four limbs affected with SPS.

## 1. Introduction

Swimming puppy syndrome—also known as swimmer syndrome, flat pup syndrome, splay leg (paraparesis), splay weak (tetraparesis), and myofibrillar hypoplasia—is one of the musculoskeletal disorders in puppies [1]. In the initial weeks of life, newborn puppies seem normal: they gain weight quickly, suck well, and appear to be completely healthy. Signs begin to appear when the puppy learns to walk (2nd-3rd week), with spreadout legs like a swimmer [1]. In some cases, there are additional complications, because such puppies tend to lie on their bellies most of the time. The center of gravity is shifted to the chest, while the soft ribs cannot maintain their correct shape; thus the chest, under the pressure of body weight, splays on both sides, and the thorax becomes flat (funnel chest) [2–4]. Some puppies show snake-like or walrus-swimming movements, crawling on their bellies with limbs extended and exorotated. Sterna concave, dorsoventral flattening of the chest, or pectus excavatum will present when forelimbs are affected [2, 3]. In cases of pectus excavatum, puppies show respiratory insufficiency, with dyspnoea, mouth continuously open, and bluish mucous membranes [3, 4]. The differential diagnosis of this disease includes encephalomeningitis, canine distemper, toxoplasmosis, neosporosis, myopathies, and spina bifida [4, 5]. The treatment success rate is dependent on the time of diagnosis and treatment [4, 5]. Usually puppies with this disease recover well after early diagnosis and treatment such as limb realignment, bandages, and physical rehabilitation [4, 5]. 

However, little is known about the prevalence of swimming puppy syndrome, other than from observational experience and a limited amount of information available on the internet (based on a search of PubMed and Scopus databases). Also, several of these publications were in the format of case reports in which only a few puppies showed signs of this disease [1–6]. From the existing information [5], it can be concluded that some of the predisposing causes of the disease are small breeds (Dachshund and Yorkshire Terrier) and also breeds with a large thorax and short limbs (Pekingese, Basset Hound, French, and English Bulldogs). Other predisposing causes [4] include puppies of normal size at birth but with faster growth than the rest of the litter and, in particular, a low number of puppies per litter. 

In the present study, the number of cases of swimming puppy syndrome was recorded by breed, together with other factors; statistical analysis was used to study the prevalence, incidence, and risk factors of this disease.

## 2. Materials and Methods

### 2.1. Animals

**Figure 1:**
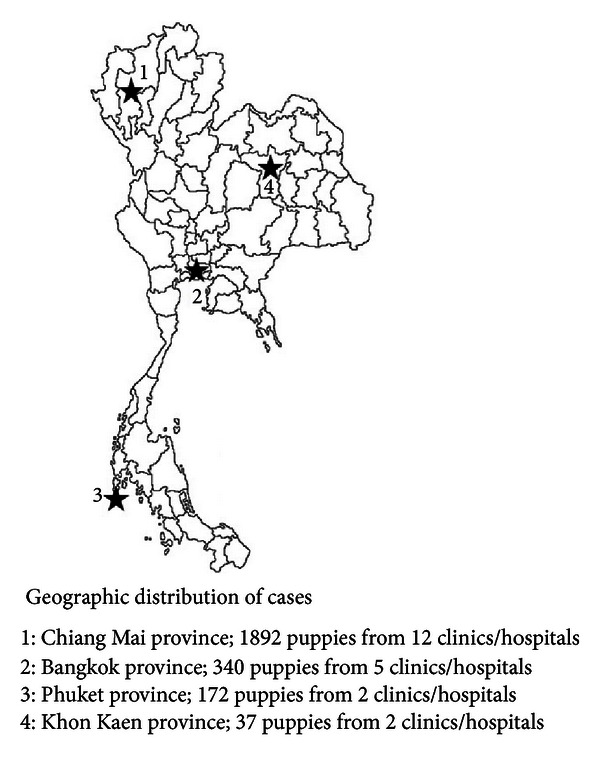
Geographic distribution of cases and clinics/hospitals included in the study in Thai.

In this retrospective study of clinical records (Table 1) of 2,443 puppies (1,183 males and 1,260 females) that were 3 months old or younger were reviewed. The puppies had undergone treatment for various reasons at 19 animal clinics/hospitals in Thailand (Figure 1) from October 2006 to September 2012.

The data collected included breed, age, weight, sex, number of puppies per litter, and floor condition on which the puppies were fostered. Moreover, the findings of the clinical examination of all affected puppies were reviewed, including affected limbs and the occurrence of pectus excavatum.

In case there was missing data, the authors called the pet owner to ask for the necessary information. If the pet owner was unable to supply the missing data, or when information about other puppies in the same litter could not be obtained, the puppy was excluded from the study. 

### 2.2. Statistical Analysis

Demographic data of the samples were described by descriptive statistics. Sex, breed size, floor conditions, and number of puppies per litter were assumed to be the associated risk factors for swimmer puppy syndrome and were investigated to determine the correlation between these factors and swimming puppy syndrome. The correlation between affected limb(s) and the occurrence of pectus excavatum was analyzed using the R statistical software program. For statistical analysis, dogs were categorized according to weight into three groups: large (>25 kg), medium (10–25), and small (<10 kg) breeds. The epi2x2 function in the epibasix package was used to examine the correlation between sex and the occurrence of the disease. A chi-squared test was used to evaluate the correlation between breed size, type of floor, and occurrence of the disease. Fisher's exact test was used to determine the correlation between affected limbs and the occurrence of pectus excavatum. Finally, the mean number of puppies per litter and the presence of diseased and nondiseased animals were analyzed by a *t*-test. The significance level was set at *P* < 0.05.

## 3. Results

The present study found that the disease started to present beginning at week 3 ± 1, but the owner brought puppy to the visiting veterinarian at week 7 ± 3.

### 3.1. Breed

A total of 2,443 puppies (1,183 males and 1,260 females) were included in this study. Twenty-two breeds of puppies were recorded in the clinical notes and of these, swimming puppy syndrome was recorded in 15 breeds. Comparing within breed, English Bulldog was found to have the highest percentage of diseased puppies, 8.33% (4/48); second was French Bulldog, 7.54% (4/53), and third was Pekingese, 6.89% (4/58). The other breeds were found to have a mean prevalence of 2.65% (range 0.78–5.55%), as shown in Table 2.

Comparison of all affected breeds revealed that Golden Retriever had the highest percentage of affected puppies (15.38%), followed by Siberian Husky (13.46%) and Labrador Retriever (9.62%). The lowest prevalence was found in Dachshunds (1.92%), followed by five other breeds, including Chihuahua, Pomeranian, Pug, Yorkshire Terrier, and Thai Bangkaew, all with the same percentage (3.85%).

 Affected puppies were compared within the same sized groups; medium-size puppies were found to have the highest number of affected animals (3.32%). Affected percentages of large and small sizes were 2.44% and 1.54%, respectively. Comparatively, the number of affected puppies in terms of breed size was not significantly different (*P* = 0.07). 

### 3.2. Sex

As shown in Table 2, of the 52 number of puppies affected by swimming puppy syndrome, 56% was males (*n* = 29), and 44% was females (*n* = 23). The prevalence of swimming puppy syndrome was not significantly different between male and female dogs (*P* = 0.484) (Table 3).

### 3.3. Number of Puppies per Litter

This study found a significant decrease (*P* < 0.01) in the number of puppies per litter in the affected group (1.92 ± 1.12) compared to clinical unaffected dogs (3.64 ± 2.24) (Table 4). The number of puppies per litter in healthy small breeds was 3 ± 1 dogs, while in affected puppies it was 1 ± 1 dogs. In medium breeds, there were 5 ± 3 healthy puppies per litter and 2 ± 1 affected puppies per litter. Large breeds showed a similar result, with 6 ± 3 healthy puppies per litter versus 3 ± 1 affected puppies per litter (Table 5).

### 3.4. Nest Floor

Swimming puppy syndrome was found to be the highest among puppies raised on concrete floors, followed by tile and wood floors, respectively (Table 6). The percentages of the disease present in animals raised on tile; wood and concrete floors were similar: 2.25%, 2.64% and 1.28%, respectively. However, no significant difference (*P* = 0.224) was established in the prevalence of swimming puppy syndrome between the different floor types. 

### 3.5. Pectus Excavatum

Eight puppies with swimming puppy syndrome had pectus excavatum. Puppies affected in all limbs with swimming puppy syndrome showed a significantly higher (*P* < 0.01) prevalence of pectus excavatum (87.5%). When only the forelimbs were affected, 20% showed signs of pectus excavatum; no signs of pectus excavatum were found when only the hindlimb was affected (Table 7).

## 4. Discussion 

Despite numerous investigations, the etiology and pathogenesis of swimming puppy syndrome are poorly understood [1, 4, 5]. Moreover, previous publications have reported that swimming puppy syndrome is an uncommon developmental abnormality observed in puppies [5, 6]. However, the present study established a prevalence (52) of 2.1% of 2,443 puppies. According to previous investigations [5–9], the etiopathogenesis of this disease is unclear; the authors propose that nutritional, neurological, hereditary, and orthopaedic causes are possible underlying factors. The results from the present study further clarify some of the unclear underlying causes of this disease.

From our observations, body weight has a significant effect on the likelihood of disease presentation. However, we did not weigh all puppies (diseased and nondiseased) at all ages to conduct a thorough statistical analysis. Another limitation of this study is that puppies were not brought to a clinic/hospital immediately upon presenting the disease. In most cases, the owner delayed bringing the pet to a clinic/hospital until week 7 ± 3 of age, while the disease typically presents at week 3 ± 1. For this reason, puppies could not be weighed at a young enough age to determine whether weight was one of the primary risk factors of this disease. However, based on veterinarians' observations, more than 90% of the affected puppies were overweight and were typically larger than puppies of the same breed at the same age. The primary focus of the present study was on the number of puppies per litter and its effect on presentation of the disease. We also documented that a lower number of puppies per litter resulted in higher body weight of puppies. This study found that a significantly (*P* < 0.01) lower number of puppies per litter were observed in the case of affected puppies (1.92 ± 1.12) compared to unaffected puppies (3.64 ± 2.24). Therefore, the statements as mentioned above can conclud that lower number of puppies per litter might be associated with higher body weight of the puppies, but this is not examined.

The results of this study indicate the possibility that hereditary or genetic factors are not the underlying cause of this disease, since out of 52 diseased puppies in this study only 2 puppies (Siberian Husky) were from the same litter. Also, their parents had never given birth to puppies with this syndrome before (in 3 previous litters). Information from other owners also indicated that the puppies affected with the disease were the first that had been born to a particular set of parents. For this reason, we believe that the disease is not genetically related. However, to fulfill the data and elucidate the possible genetic basis of swimming puppy syndrome, our group is conducting a molecular genetics study of candidate genes for this disease; the results are to be published in the near future. 

In the case of pigs, splay leg has been proven to be a hereditary disorder [10, 11]. Maak and others [11] pinpointed the candidate genes for splay leg in piglets using DNA microarray data, comparing the genome-wide gene expression of three hindlimb muscles between affected and healthy piglets. They found 63 transcripts with differences in two muscle groups and 5 gene differences in three muscle groups. Based on their study results, they concluded that certain genes were associated with splay leg in piglets and that future studies of the genetic mechanism needed to be performed in order to achieve a better understanding of the pathogenesis of this disease. 

The present study found that the disease started to present beginning at week 3 ± 1, when normal puppies are learning to stand and walk, that is, after 10–14 days of age [8, 12]. Previous reports also found that the disease started in the 2nd to 4th week [5, 13]. However, in this study, owners typically did not bring their puppies to a clinic or hospital when the disease first presented, but instead waited until puppies were 7 ± 3 weeks of age. Almost none of the owners recognized that the swimming movement was an abnormality. This is responsible for the delay in treatment, which leads to a reduction in the success rate of treatment. All previous reports agree that early diagnosis and treatment will result in a good prognosis, while delayed treatment results in poor prognosis.

A surprising result from this study, when comparing the 52 affected puppies, was that Golden Retrievers (15.38%) had the highest number of puppies affected with swimming puppy syndrome, followed by Siberian Husky (13.46%) and Labrador Retriever (9.62%). Moreover, we also found that many medium breeds (Bulldog, French Bulldog, Crossbreed, and Thai Bangkaew) were affected by this disease. However, these figures could be explained because those medium and large breeds that were more affected by swimming puppy syndrome had a small number of average puppies per litter (3 ± 1 and 2 ± 1 puppies per litter for large and medium breeds, resp.). This low number of puppies per litter is a cause of overweight puppies, to the extent where puppies may not be able to stand and walk properly. However, when comparing the number of affected puppies within a breed, the highest percentage of disease was found in Bulldogs (8.33%), French Bulldogs (7.54%), and Pekingese (6.89%), findings which were in agreement with previous reports [4, 5, 9, 12] where swimming puppy syndrome was commonly found in brachycephalic and chondrodystrophoid breeds. 

Some clinicians have discussed the effects of the nest floor on the prevalence of this disease, but no scientific research on this issue has yet been published. In this study, tile floors produced the highest number (56%) of cases, followed by concrete floors (29%) and wood floors (15%); however, there was no significant difference (*P* > 0.05) between the types of floor.

Affected limb (pathological limb) is a topic which has been widely discussed in the literature concerning this disease. The present results found that in 75% of cases the affected limb was predominantly a hindlimb; a lower number of cases (15.38%) were found to have all four limbs affected; the lowest number represented cases where only the forelimb was affected (9.62%). Together with an affected limb, pectus excavatum was found in 8 puppies: 7 puppies with all limbs affected and 1 puppy with a hindlimb affected. Only 1 puppy with all limbs affected did not show signs of pectus excavatum. Because of continuous sternal recumbency, particularly in puppies affected in the forelimb, the pressure of the body weight against the sternum causes flattening of the chest. The study data showed that an affected forelimb was significantly related to the presence of pectus excavatum. This was in agreement with previous case reports [3–5, 13, 14], which found that all puppies with an affected forelimb also had pectus excavatum. 

One disadvantage of this study is that we could not conclude that swimming puppy syndrome is one of the risk factors of pectus excavatum, because this study did not record the number of puppies affected only with pectus excavatum, without swimming puppy syndrome. A search of previous publications revealed that the prevalence of pectus excavatum in dogs has not been reported; however, in cats it was found to be 2.05% (5/244) [15] and in humans 1.0–1.27% [16, 17]. Although the present study did not primarily focus on the incidence of pectus excavatum, 0.33% of puppies (8 out of 2,443) were found to have pectus excavatum. Compared with the previous study, it seems possible that a higher percentage of cats are affected by pectus excavatum. 

Swimming puppy syndrome has been considered to be uncommon, based on clinical observations and a low number of published reports. But our study indicated that a few number of puppies are affected by this disease. The primary risk factor of this disease is a lower number of puppies per litter, while other factors, including sex and floor type, may not be involved. The present results could be applied for use in future studies: for example, the possible genetic control of this disease and the potential involvement of other risk factors. Moreover, all limb lesions are associated with pectus excavatum. 

## Figures and Tables

**Table 1:** Number of puppies in each breed included in the study.

Breed	Total	Male	Female	Number of litters	Number of puppies/litter (mean ± SD)
Small breed (<10 kg)					
Chihuahua	256	125	131	111	2 ± 1
Dachshund	18	8	10	5	4 ± 2
Jack Russell Terrier*	19	15	4	6	3 ± 1
Pekingese	58	27	31	22	3 ± 1
Pomeranian	293	131	162	130	2 ± 1
Poodle	82	45	37	26	3 ± 1
Pug	77	31	46	23	3 ± 1
Shih Tzu	265	123	142	76	3 ± 1
Yorkshire Terrier	165	74	91	79	2 ± 1
Medium breed (10–25 kg)					
Beagle*	60	36	24	13	5 ± 2
Bulldog (English)	48	25	23	14	3 ± 2
French Bulldog	53	23	30	14	4 ± 1
Crossbreed	122	48	74	21	6 ± 4
Shetland Sheepdog*	11	5	6	2	6 ± 1
Thai Bangkaew	85	49	36	13	7 ± 2
Thai Ridgeback*	13	5	8	2	7 ± 2
Large breed (25–40 kg)					
American Pit Bull Terrier*	71	32	39	9	8 ± 2
German Shepherd*	76	42	34	11	7 ± 2
Golden Retriever	217	103	114	36	6 ± 3
Labrador Retriever	213	115	98	36	6 ± 2
Rottweiler*	109	48	61	16	7 ± 3
Siberian Husky	132	73	59	28	5 ± 2

Total	2,443	1,183	1,260	=	—

*Breed was not affected with swimming puppy syndrome.

**Table 2:** Number of puppies in different breeds affected with swimming puppy syndrome.

	Healthy puppies			Swimming puppy syndrome			%**
	Male	Female	Total	Male	Female	Total (%*)	
Small breed (<10 kg)							
Chihuahua	125	131	256	1	1	2 (0.78)	3.85
Dachshund	8	10	18	1	—	1 (5.55)	1.92
Pekingese	27	31	58	1	3	4 (6.89)	7.69
Pomeranian	131	162	293	1	1	2 (0.68)	3.85
Poodle	45	37	82	2	1	3 (3.65)	5.77
Pug	31	46	77	—	2	2 (2.59)	3.85
Shih Tzu	123	142	265	1	2	3 (1.17)	5.77
Yorkshire Terrier	74	91	165	1	1	2 (1.21)	3.85
Medium breed (10–25 kg)							
Bulldog (English)	25	23	48	2	2	4 (8.33)	7.69
French Bulldog	23	30	53	1	3	4 (7.54)	7.69
Crossbreed	48	74	122	3	—	3 (2.45)	5.77
Thai Bangkaew	49	36	85	2	1	2 (2.35)	3.85
Large breed (25–40 kg)							
Golden Retriever	103	114	217	6	2	8 (3.68)	15.38
Labrador Retriever	115	98	213	3	2	5 (2.34)	9.62
Siberian Husky	73	59	132	4	3	7 (5.30)	13.46

Total	1,000	1,084	2,084	29	23	52	100

*Percentage of affected puppies compared within breeds.

**Percentage of affected puppies compared between breeds.

**Table 3:** Factor of sex on expression of disease.

Sex	Total	Disease present (cases)	Disease absent (controls)
Male	1,184	29 (2.45%)	1,155 (97.55%)
Female	1,259	23 (1.83%)	1,236 (98.17%)

Pearson's chi-squared statistic (includes Yates' continuity correction) = 0.423; *P* value using Fisher's exact test (1 degree of freedom) = 0.484; estimate of odds ratio = 1.248; 95% confidence limits for true odds ratio = [0.72, 2.166]; estimate of risk difference (*p*_1_ − *p*_2_) in case-control studies = 0.055; 95% confidence limits for risk difference = [0.006, 0.105].

**Table 4:** Number of puppies per litter with presence or absence of disease.

Data	Disease present (cases)	Disease absent (controls)
Number of samples	52	641
Mean number of puppies per litter	1.92	3.65
Standard deviation	1.12	2.24

*t* = 9.6906; degrees of freedom = 88.859; *P* < 0.001.

**Table 5:** Number of puppies per litter in normal and affected groups.

Breed	Number of puppies per litter		*P* value
	Disease absent	Disease present	
Small breed			
Chihuahua	2 ± 1	1 ± 0	0.000
Dachshund	4 ± 2	—	—
Jack Russell Terrier	3 ± 1	—	—
Pekingese	3 ± 1	1 ± 1	0.001
Pomeranian	2 ± 1	2 ± 0	0.001
Poodle	3 ± 1	1 ± 0	0.000
Pug	3 ± 1	2 ± 1	0.001
Shih Tzu	3 ± 1	1 ± 1	0.006
Yorkshire Terrier	2 ± 1	1 ± 0	0.000
Mean ± SD	**3** ± **1**	**1** ± **1**	
Medium breed			
Beagle	5 ± 4	—	—
Bulldog (English)	3 ± 2	2 ± 1	0.002
French Bulldog	4 ± 1	2 ± 1	0.008
Crossbreed	6 ± 4	1 ± 1	0.000
Shetland Sheepdog	6 ± 1	—	—
Thai Bangkaew	7 ± 2	4 ± 1	0.005
Thai Ridgeback	7 ± 2	—	—
Mean ± SD	**5** ± **3**	**2** ± **1**	
Large breed			
American pit Bull Terrier	8 ± 2	—	—
German Shepherd	7 ± 2	—	—
Golden Retriever	6 ± 3	2 ± 1	0.000
Labrador Retriever	6 ± 2	3 ± 1	0.000
Rottweiler	7 ± 3	—	—
Siberian Husky	5 ± 2	3 ± 2	0.011
Mean ± SD	**6** ± **3**	**3** ± **1**	

**Table 6:** Number of litters on different floor types.

	Floor type			Total
	Tile	Concrete	Wood	
Disease present (cases)	29 (2.25%)	15 (2.64%)	8 (1.28%)	52
Disease absent (controls)	1,261 (97.75%)	554 (97.36%)	617 (98.72%)	2,432

Total	1,290	569	625	2,484

Chi squared = 2.9861; degrees of freedom = 2; *P* value = 0.2247.

**Table 7:** Relationship between affected limb and occurrence of pectus excavatum.

Affected limb	Pectus excavatum		Total
	Present	Absent	
Forelimb	1 (20%)	4 (80%)	5
All limbs	7 (87.5%)	1 (12.5%)	8
Hindlimb	—	39 (100%)	39

Total	8 (15.38%)	44 (84.62%)	52

*P *< 0.001.
